# A developed serum-free medium and an optimized chemical cocktail for direct conversion of human dermal fibroblasts into brown adipocytes

**DOI:** 10.1038/s41598-020-60769-x

**Published:** 2020-02-28

**Authors:** Yukimasa Takeda, Ping Dai

**Affiliations:** Department of Cellular Regenerative Medicine, Graduate School of Medical Science, Kyoto Prefectural University of Medicine, 465 Kajii-cho, Kawaramachi-Hirokoji, Kamigyo-ku, Kyoto, 602-8566 Japan

**Keywords:** Cell biology, Stem cells

## Abstract

Brown adipocytes coordinate systemic energy metabolism associated with the pathogenesis of obesity and related metabolic diseases including type 2 diabetes. We have previously reported chemical compound-induced brown adipocytes (ciBAs) converted from human dermal fibroblasts without using transgenes. In this study, to reveal a precise molecular mechanism underlying the direct conversion and human adipocyte browning, we developed serum-free brown adipogenic medium (SFBAM) with an optimized chemical cocktail consisting of Rosiglitazone, Forskolin, and BMP7. During the direct conversion, treatment with BMP7 enhanced *Ucp1* expression rather than the conversion efficiency in the absence of BMP signalling inhibitors. Moreover, treatment with a TGF-β signalling pathway inhibitor was no longer required in the serum-free medium, likely because the TGF-β pathway was already suppressed. SFBAM and the chemical cocktail efficiently converted human dermal fibroblasts into ciBAs within four weeks. The ciBAs exhibited increased mitochondrial levels, elevated oxygen consumption rate, and a response to β-adrenergic receptor agonists. Thus the ciBAs converted by the serum-free medium and the chemical cocktail provide a novel model of human brown (beige) adipocytes applicable for basic research, drug screening, and clinical applications.

## Introduction

Direct lineage reprogramming is an attractive approach to rapidly prepare desired cell types from patient-derived autologous somatic cells for transplantation therapy bypassing a pluripotent state^1,2^. So far human fibroblasts have been directly converted into myoblasts, cardiomyocytes, hepatocytes, and neurons by overexpression of a specific set of transcription factors^3–6^. The fibroblasts could also be converted to brown adipocytes by overexpression of two transcription factors, C/EBP-β and C-Myc^7^. However, in general direct reprogramming with virus vector-mediated overexpression of multiple transcription factors requires complicated procedures and also increases the risk of genomic instability and subsequent tumor formation^8^. Accumulating evidence has suggested that small molecules enable efficient cellular differentiation and direct reprogramming by modulating activity of target transcription factors as well as major signalling pathways^9,10^. We have previously reported that neuronal cells and brown adipocyte-like cells were converted from human fibroblasts by small molecules only without the use of transgene^11,12^. In addition, combinations of small molecules only also enable conversion of human fibroblasts into astrocytes, Schwann cells, osteoblasts, and cardiomyocytes^13–16^. Such simple and reproducible techniques for direct reprogramming are promising for the preparation of target cells with a reduced risk of mutations and tumorigenesis for transplantation therapy and other clinical applications^1^.

Two types of adipocytes, white and brown adipocytes, regulate energy storage and expenditure, respectively^17^. Brown adipocytes possess thermogenic properties mediated by Uncoupling protein 1 (UCP1) localized in the inner mitochondrial membrane. Recent studies have revealed that metabolically active beige adipocytes were generated in white adipose tissue depots by environmental stimulations such as chronic cold exposure and long-term treatment with β-adrenalin receptor agonists, which is so-called adipocyte browning^18^. Classical brown adipocytes and beige adipocytes share morphological characteristics such as multilocular lipid droplets and enriched mitochondria, although the thermogenic capacity dependent on UCP1 and its expression are inducible and reversible in beige adipocytes. Therefore, the manipulation of beige adipocyte population and adipocyte browning has a therapeutic potential for the treatment of obesity-associated metabolic diseases including type 2 diabetes and cardiovascular diseases. Since human beige adipocytes are sporadically dispersed in subcutaneous white adipose tissue depots^19^, it is not feasible to isolate a sufficient amount of homogeneous beige adipocytes from different human specimens^20^. A cell model of human beige adipocytes has been in demand for better understanding of the molecular mechanism underlying adipocyte browning as well as identification of small molecules enhancing the population of beige adipocytes in our body.

We have previously reported chemical compound-induced brown adipocytes, ciBAs, by the combination of five chemical compounds, 5CD-GM, which consists of SB-431542, a TGF-β signalling inhibitor, LDN-193189 and Dorsomorphin, BMP signalling inhibitors, and Forskolin, a cAMP inducer, in addition to Rosiglitazone, a PPARγ agonist^12^. The molecular mechanism underlying the direct conversion driven by these compounds remains largely unclear, although understanding of it is essential for a better conversion protocol for ciBA and other cell types. However, animal serum includes abundant bioactive materials and animal-derived contaminants which modulate cellular signalling pathways including TGF-β and BMP pathways that play a critical role in the chemical conversion. Furthermore, unknown compositions and high batch-to-batch variation could disturb reproducibility and reliability of the preparation of ciBAs for clinical applications as well as ciBA-based drug screening. Therefore, we developed serum-free brown adipogenic medium along with the optimization of the chemical cocktail for the direct conversion. The ciBAs simply induced by the serum-free medium might be a novel model for human brown (beige) adipocytes.

## Results

### BMP7 treatment enhanced *Ucp1* expression in the condition without BMP signalling inhibitors

**Figure 1:**
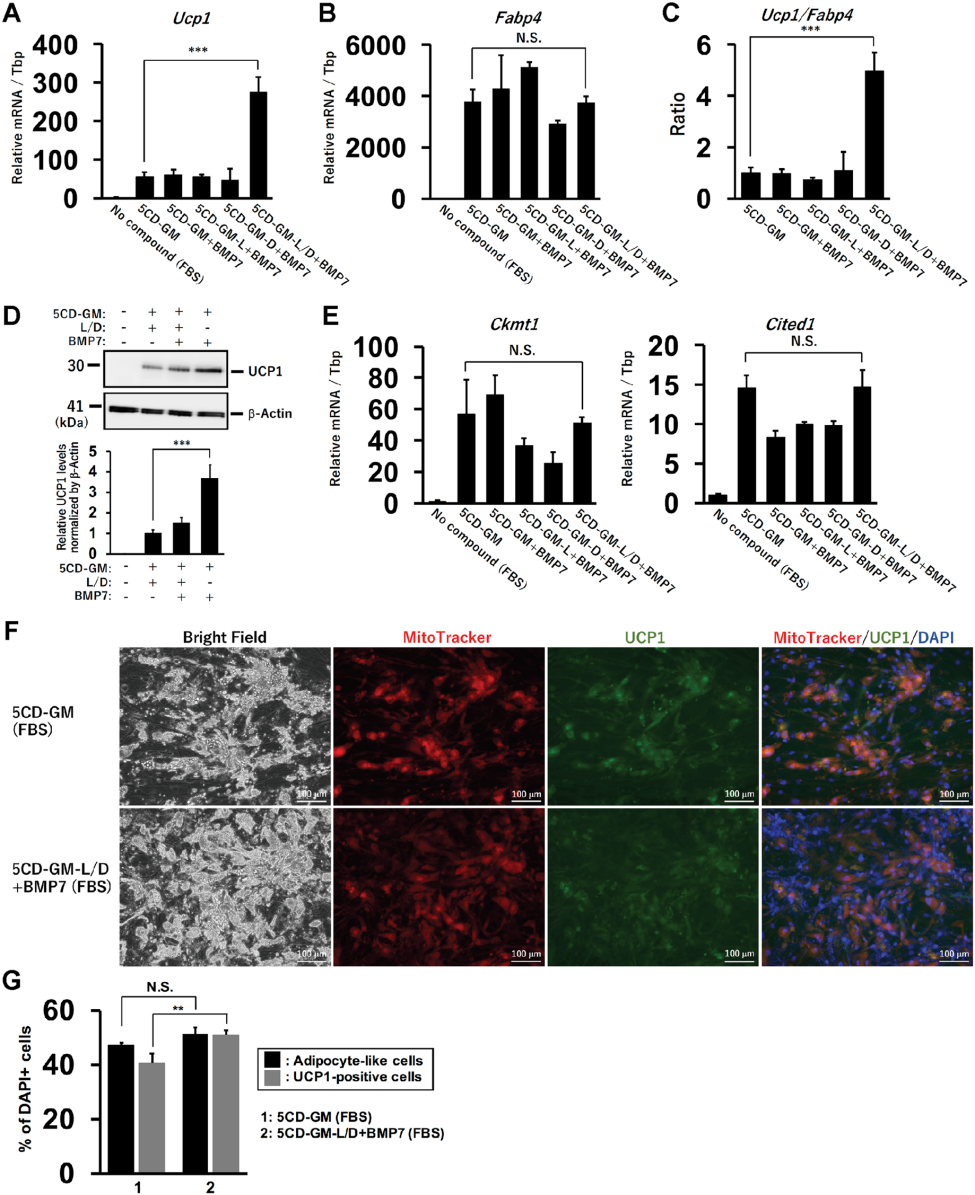
BMP7 treatment enhances *Ucp1* expression in the chemical compound-induced brown adipocytes (ciBAs) from human dermal fibroblasts. (**A,B**) The expression of *Ucp1* (**A**) and *Fabp4* (**B**) was quantified by qRT-PCR. Human dermal fibroblasts were treated with BMP7 and the chemical cocktail, 5CD-GM, in the FBS-containing adipogenic medium. Either or both of BMP signalling inhibitors, LDN-193189 (L) and Dorsomorphin (D) were removed from the 5CD-GM as indicated. (**C**) The ratio of *Ucp1* to *Fabp4* expression was calculated to evaluate a brown phenotype under each experimental condition. (**D**) UCP1 protein levels were quantified by western blotting analysis. The band intensities were quantified by densitometry using ImageJ software. β-Actin was used as a loading control for normalization. (**E**) qRT-PCR analyses of other human brown adipocyte-specific genes, *Ckmt1* and *Cited1*. (**F**) Representative images of bright field, mitochondrial labelling with MitoTracker (red), UCP1 protein expression (green), and merged image in ciBAs induced by either 5CD-GM or 5CD-GM-L/D+BMP7 in the FBS-containing medium. The nuclei were visualized by DAPI (blue). Scale bars represent 100 μm. (**G**) To evaluate the conversion efficiency, the percent ratio of adipocyte-like cells with lipid droplets and UCP1-positive cells were calculated. Data represent mean ± SD. Student’s t-test: **P < 0.01, ***P < 0.001, N.S.; not significant.

We have previously reported chemical compound-induced brown adipocytes (ciBAs) from human dermal fibroblasts^12^. The direct conversion into ciBAs was performed by the combination of five small molecules, Rosiglitazone, Forskolin, SB-431542, LDN-193189, and Dorsomorphin, which was referred to as “5CD-GM”. In order to optimize the chemical combination and experimental conditions, an original adipogenic medium was newly prepared for the direct conversion. Human dermal fibroblasts (HDF38) were incubated with the adipogenic medium containing 10% FBS for 3 weeks. The treatment with 5CD-GM induced the expression of *Ucp1*, a brown adipocyte-specific marker, compared to that in control cells treated with the medium only (Fig. 1A). BMP signalling pathway activated by BMP7 has been known to be involved in rodent brown adipogenesis^21,22^, however the requirement for the direct conversion has remained unknown. The effect of BMP7 was examined in the presence or absence of the BMP signalling inhibitors, LDN-193189 (L) and Dorsomorphin (D), which are included in the combination of 5CD-GM. BMP7 treatment was not effective under the treatment with either one or both of the inhibitors, however, *Ucp1* expression was increased specifically in the absence of both the inhibitors from the combination, which is represented by 5CD-GM-L/D+BMP7. The expression of *Fabp4*, an adipocyte enriched gene, was not significantly changed in the treatment with BMP7 (Fig. 1B). Adipocyte browning was evaluated by the ratio of the expression of *Ucp1* to *Fabp4*^23^. The ratio was increased by BMP7 treatment specifically under the condition without both the inhibitors (Fig. 1C). Western blotting results showed that UCP1 protein expression was enhanced in the condition of 5CD-GM-L/D+BMP7 compared to that in 5CD-GM condition (Fig. 1D). The expression of other human brown adipocyte-specific genes, *Ckmt1* and *Cited1*, was not significantly changed between these conditions (Fig. 1E), suggesting that BMP7 mainly has a unique effect on the activation of *Ucp1* expression rather than promoting the direct conversion into ciBAs.

Immunocytochemical analysis in ciBAs induced by the combination of 5CD-GM and 5CD-GM-L/D+BMP7 indicated that cellular lipid accumulation (leftmost panels of Fig. 1F and Supplementary Fig. S1A), mitochondria staining (left middle panels of Fig. 1F and Supplementary Fig. S1A), and UCP1 expression (right middle panels of Fig. 1F and Supplementary Fig. S1A) were well overlapped (rightmost panels of Fig. 1F and Supplementary Fig. S1A), which suggests that most adipocyte-like cells with lipid droplets had elevated mitochondria levels and UCP1 expression. The conversion efficiency was not significantly different between 5CD-GM and 5CD-GM-L/D+BMP7 (Fig. 1G), however, the treatment with 5CD-GM-L/D+BMP7 enhanced the UCP1-positive cell population compared to the ones in the treatment with 5CD-GM, consistent with the observation that BMP7 activated *Ucp1* expression rather than *Fabp4*.

### Development of serum-free brown adipogenic medium (SFBAM) for the direct conversion

**Figure 2:**
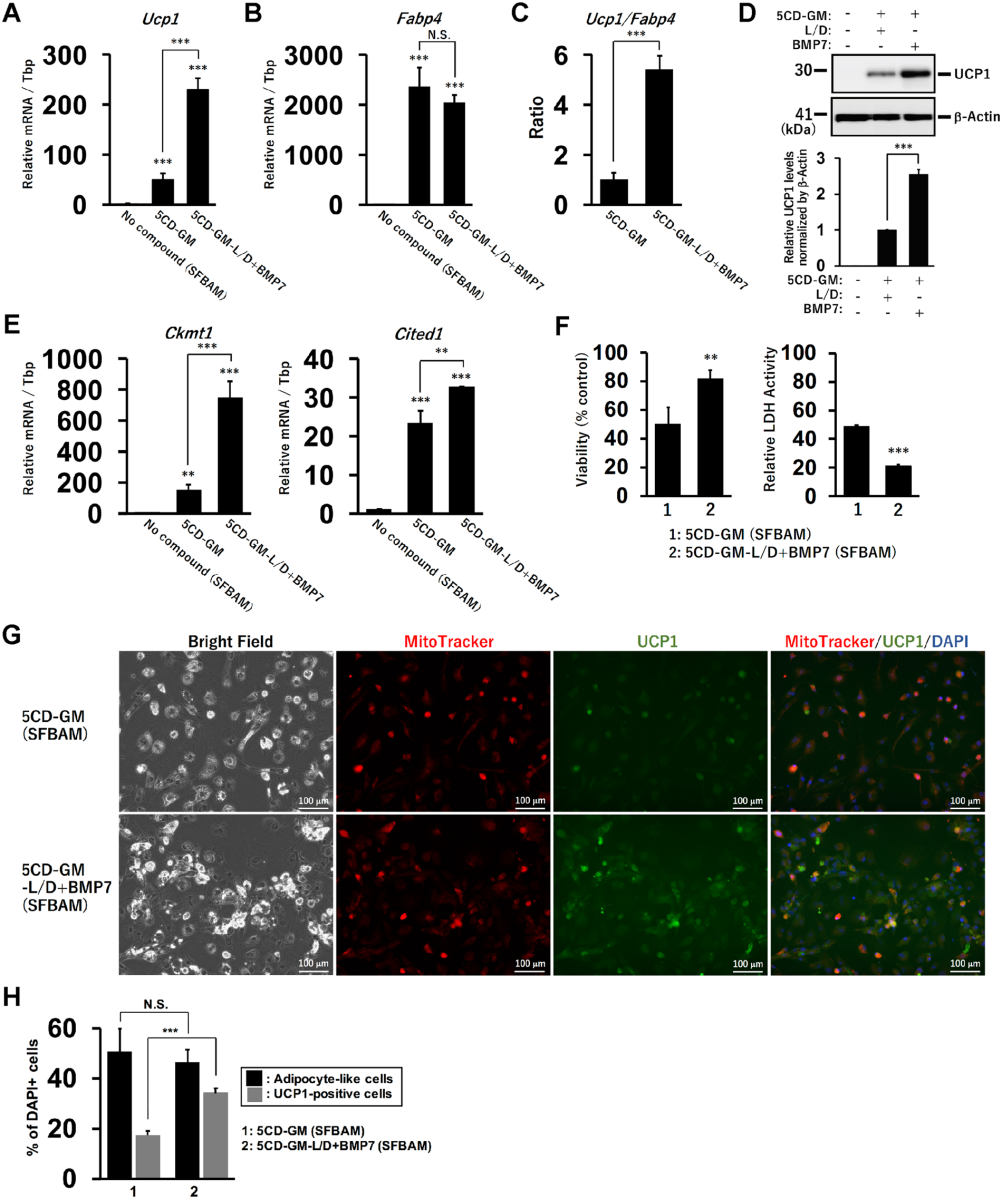
The serum-free brown adipogenic medium enables the direct conversion into ciBAs. (**A,B**) The expression of *Ucp1* (**A**) and *Fabp4* (**B**) was quantified by qRT-PCR in ciBAs induced by the combination of either 5CD-GM or 5CD-GM-L/D+BMP7 in the serum-free brown adipogenic medium (SFBAM). (**C**) The ratio of *Ucp1* to *Fabp4* expression was calculated to evaluate a brown phenotype in these ciBAs. (**D**) UCP1 protein levels were evaluated by western blotting analysis. The band intensities were quantified by densitometry. β-Actin was used as a loading control for normalization. (**E**) qRT-PCR analyses of other human brown adipocyte specific genes, *Ckmt1* and *Cited1*. (**F**) Cell viability was measured using the WST-8 reagent in ciBAs induced by either 5CD-GM or 5CD-GM-L/D+BMP7 in SFBAM. Cytotoxicity was assessed by measuring lactate dehydrogenase (LDH) activity in the culture supernatants. (**G**) Representative images of bright field, MitoTracker (red), UCP1 protein (green), and merged image in ciBAs induced by either 5CD-GM or 5CD-GM-L/D+BMP7 in SFBAM. The nuclei were visualized by DAPI (blue). Scale bars represent 100 μm. (**H**) To evaluate the conversion efficiency, the percent ratio of adipocyte-like cells and UCP1-positive cells was calculated. Data represent mean ± SD. Student’s t-test: **P < 0.01, ***P < 0.001, N.S.; not significant.

FBS contains abundant bioactive metabolites, cytokines, lipids, exosome, and lot-to-lot variations which affect cellular signalling pathways and broad patterns of gene expression. To define experimental conditions for the direct conversion, next we tried to remove FBS from the adipogenic medium by substituting specific fatty acids-binding albumin for FBS (Table 1). The albumin binding linoleic acid and oleic acid is generally beneficial for cell proliferation as a source of fatty acids. The fibroblasts were incubated with the serum-free brown adipogenic medium (SFBAM) including the combination of either 5CD-GM or 5CD-GM-L/D+BMP7 for 3 weeks. Both the conditions induced the expression of *Ucp1* and *Fabp4*, suggesting that SFBAM also converted human fibroblasts into adipocyte-like cells with a feature of brown adipocytes (Fig. 2A,B). Consistent with the observation in the FBS-containing medium, BMP7 treatment in the absence of the BMP inhibitors further enhanced the expression of *Ucp1*, but not *Fabp4*. The ratio of *Ucp1* to *Fabp4* expression was increased by the treatment with BMP7 without the BMP inhibitors (Fig. 2C). According to the elevated *Ucp1* mRNA level, the protein level was also increased by BMP7 (Fig. 2D). The expression of other human brown adipocyte markers, *Ckmt1* and *Cited1*, was also elevated by the treatment with BMP7 (Fig. 2E). The UCP1 protein levels were comparable in ciBAs induced by the FBS-containing and serum-free conditions (Supplementary Fig. S2A).

**Table 1:** The components of serum-free brown adipogenic medium (SFBAM) for the direct conversion to ciBAs from human dermal fibroblasts.

Components	Concentration
Triiodothyronine	0.1 μM
Dexamethasone	0.5 μM
IBMX	0.03 mM
Insulin	6.6 μg/ml
L-ascorbic acid 2-phosphate	100 μg/ml
Linoleic acid, Oleic acid-Albumin	1 mg/ml
Penicillin/Streptomycin	100 units/ml

Cell viability was increased in ciBAs induced by 5CD-GM-L/D+BMP7 compared with ciBAs induced by 5CD-GM in SFBAM (Fig. 2F). In contrast cytotoxicity evaluated by release of lactate dehydrogenase (LDH) into the culture supernatants was reduced, suggesting that the removal of the two BMP signalling inhibitors was beneficial to reduce the cytotoxicity during the conversion. Immunocytochemical analysis demonstrated that the adipocyte-like cells with lipid droplets (leftmost panels of Fig. 2G and Supplementary Fig. S1B) had elevated mitochondria signals (left middle panels of Fig. 2G and Supplementary Fig. S1B) and UCP1 staining (right middle panels of Fig. 2G and Supplementary Fig. S1B). In addition, these signals well overlapped one another (rightmost panels of Fig. 2G and Supplementary Fig. S1B), suggesting that the adipocyte-like cells could also be considered as ciBAs. The efficiency of the conversion was not different between these conditions (Fig. 2H), but the population of UCP1-positive cells was increased in the condition of 5CD-GM-L/D+BMP7.

### An opposite effect of TGF-β signalling inhibitor on the direct conversion into ciBA between the FBS-containing and -free medium

**Figure 3:**
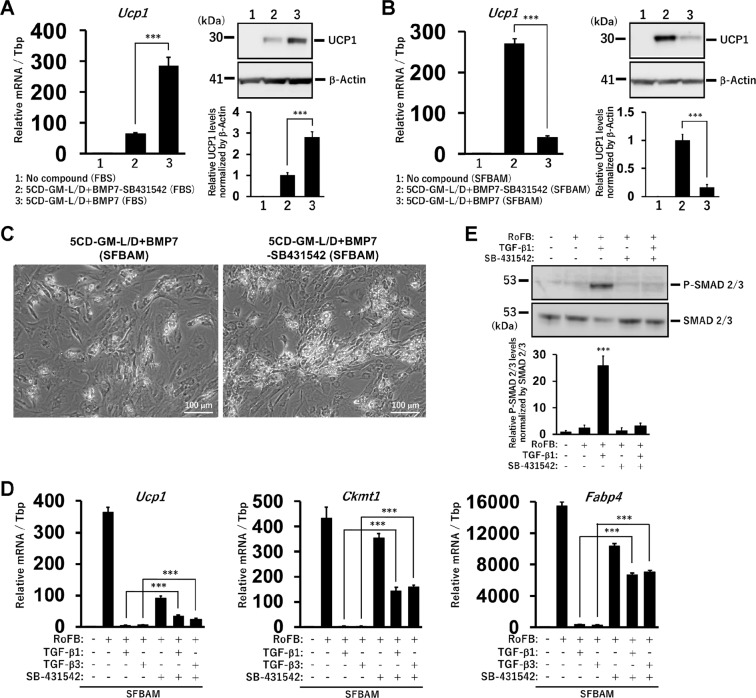
TGF-β signalling pathway inhibitor, SB-431542, is dispensable for the conversion of ciBAs in the serum-free medium. (**A**) *Ucp1* expression was quantified by qRT-PCR in ciBAs induced by the combination of 5CD-GM-L/D+BMP7 either with or without SB-431542 in the FBS-containing medium. UCP1 protein levels were quantified by western blotting analysis. β-Actin is a loading control. (**B**) *Ucp1* expression was quantified by qRT-PCR in ciBAs induced by the same combinations in SFBAM. UCP1 protein levels were quantified by western blotting analysis. (**C**) Bright-field images of ciBAs induced by the combinations either with or without SB-431542 in SFBAM. Scale bars represent 100 μm. (**D**) During the conversion to ciBAs with the combination of RoFB (Rosiglitazone, Forskolin, and BMP7) in SFBAM, a TGF-β ligand, either TGF-β1 or TGF-β3, and SB-431542 were treated as indicated. The expression of *Ucp1*, *Ckmt1*, and *Fabp4* was quantified by qRT-PCR. (**E**) Phosphorylated-SMAD2/3 and total SMAD2/3 proteins were detected by immunoblotting. The band intensities were quantified by densitometry. Data represent mean ± SD. Student’s t-test: ***P < 0.001.

Consistent with our previous results^12^, the removal of SB-431542, an inhibitor of TGF-β signalling pathway, from the combination of 5CD-GM-L/D+BMP7 decreased both *Ucp1* mRNA and protein levels in the FBS-containing medium (Fig. 3A). However, in ciBAs induced by using SFBAM, the removal of SB-431542 unexpectedly enhanced *Ucp1* expression (Fig. 3B). Consistent with the observation, bright-field images suggested that the population of adipocyte-like cells was enhanced due to the removal of SB-431542 in the serum-free medium (Fig. 3C). Eventually, the optimized chemical cocktail for the direct conversion with SFBAM was simply configured with Rosiglitazone (Ro), Forskolin (F), and BMP7 (B), which is designated as “RoFB” hereafter.

The activation of TGF-β signalling pathway is involved in induction of obesity-induced adipose tissue fibrosis as well as inhibition of adipocyte differentiation^24,25^. To reveal precise effects of SB-431542 on the direct conversion, two kinds of TGF-β family ligands, TGF-β1 and TGF-β3, were treated during the conversion with RoFB in SFBAM. The treatment with either TGF-β1 or TGF-β3 dramatically reduced the expression of *Ucp1*, *Ckmt1*, and *Fabp4* (Fig. 3D). The addition of SB-431542 only reduced these mRNA levels in the serum-free medium, whereas the treatment with both the TGF-β ligand and SB-431542 relatively upregulated the expressions of *Ucp1*, *Ckmt1*, and *Fabp4* compared to the ones in the treatment with either TGF-β1 or TGF-β3 only. Western blotting analysis indicates that phosphorylation of SMAD2/3 transcription factors, downstream effectors for TGF-β signalling pathway, was at a low level in the serum-free medium (Fig. 3E). The addition of TGF-β1 ligand activated the phosphorylation, while the additional treatment with SB-431542 reversed it. These results suggested that the treatment with SB-431542 was not required in the direct conversion with SFBAM.

### Requirement of each component in the chemical cocktail, RoFB, and the serum-free medium, SFBAM

**Figure 4:**
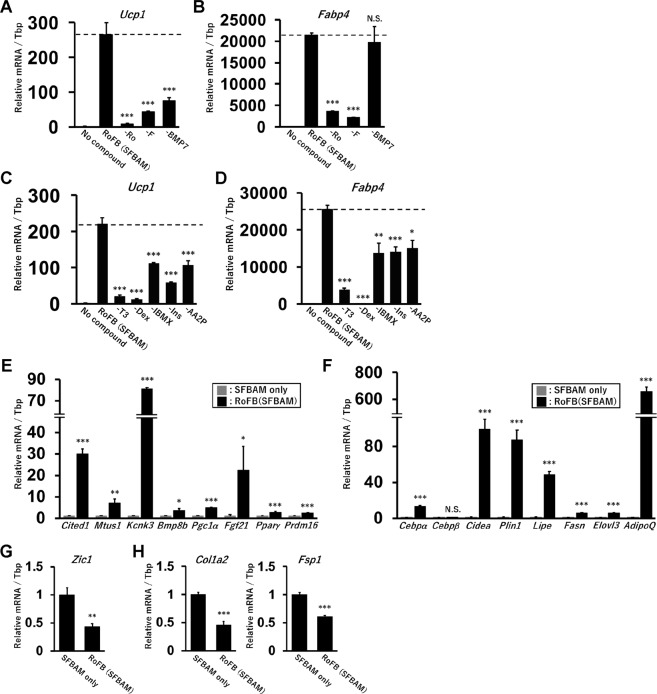
Requirement of each component consisting of the chemical cocktail and SFBAM for the ciBA conversion. (**A, B**) The expression of *Ucp1* (**A**) and *Fabp4* (**B**) was quantified by qRT-PCR in ciBAs induced by RoFB in SFBAM. Each component, Rosiglitazone (Ro), Forskolin (F), and BMP7, was removed from the combination of RoFB as indicated. (**C,D**) The expression of *Ucp1* (**C**) and *Fabp4* (**D**) was quantified in ciBAs induced by RoFB in SFBAM. Each component, T3, Dexamethasone (Dex), IBMX, insulin (Ins), and L-ascorbic acid 2-phosphate (AA2P) was removed from SFBAM as indicated. (**E, F**) The expression of human brown adipocyte marker genes (**E**) and adipocyte-enriched genes (**F**) was evaluated by qRT-PCR in ciBAs induced by RoFB in SFBAM for 3 weeks. (**G, H**) qRT-PCR analyses of zinc finger protein of cerebellum 1, *Zic1* (**G**), and fibroblast marker genes, *Col1a2* and *Fsp1* (**H**). Data represent mean ± SD. Student’s t-test: *P < 0.05, **P < 0.01, ***P < 0.001, N.S.; not significant.

To confirm requirements for the direct conversion, each of the components of RoFB was removed. *Ucp1* expression was strongly reduced in ciBAs induced by the loss of each component (Fig. 4A). The expression of adipocyte-enriched gene, *Fabp4*, was reduced in the absence of Rosiglitazone and Forskolin, but not BMP7 (Fig. 4B). Next, each of the components in SFBAM as shown in Table 1 was removed and ciBAs were induced with each medium containing RoFB. The results indicated that each of them was required for the expression of both *Ucp1* and *Fabp4* (Fig. 4C,D). In particular, the loss of T3, an agonist for thyroid hormone receptor, and Dexamethasone (Dex), an agonist for glucocorticoid receptor, strongly suppressed the expression. These results suggested that all the components in RoFB and SFBAM contributed to the direct conversion of human dermal fibroblasts into ciBAs.

Next, to develop serum-free maintenance medium, ciBAs induced with RoFB in SFBAM for 3 weeks were cultured for an additional 1 week with SFBAM from which each component was removed (Supplementary Fig. S3A,B). The absence of T3 and L-ascorbic acid 2-phosphate (AA2P) did not decrease the expression of both *Ucp1* and *Fabp4*, while the absence of Dexamethasone (Dex) and Insulin (Ins) decreased the expression, indicating that Dex and Ins were particularly required for the maintenance of ciBAs. During the maintenance step Rosiglitazone continued to be treated, because the loss of Rosiglitazone greatly reduced *Ucp1* expression, as shown in the first lane in Supplementary Fig. S3C. Then, the single component was added to DMEM-based medium only supplemented with fatty acids-binding albumin and antibiotics. The treatment with either Dex or Ins slightly restored *Ucp1* expression (Supplementary Fig. S3C), while both together further restored the expression of *Ucp1*. No other additional component in the combination of Dex and Ins increased *Ucp1* expression. Altogether, T3, IBMX, and AA2P were not necessary for the maintenance step to maintain *Ucp1* expression, so we determined the formulation for the serum-free brown adipogenic maintenance medium (SFBAMaM), as shown in Table 2.

**Table 2:** The components of serum-free brown adipogenic maintenance medium (SFBAMaM) for the maintenance of ciBAs.

Components	Concentration
Rosiglitazone	0.1 μM
Dexamethasone	0.5 μM
Insulin	6.6 μg/ml
Linoleic acid, Oleic acid-Albumin	1 mg/ml
Penicillin/Streptomycin	100 units/ml

To characterize ciBAs as human brown adipocytes, the expression of other brown/beige adipocyte-enriched genes was further examined. ciBAs showed increased expression of human beige adipocyte marker genes such as *Cited1*, *Mtus1*, *Kcnk3*, *Bmp8b*, *Pgc1α*, *Fgf21*, *Pparγ*, *Prdm16* (Fig. 4E) as well as adipocyte-enriched genes such as *Cebpα*, *Cidea*, *Plin1*, *Lipe*, *Fasn*, *Elovl3*, and *AdipoQ* (Fig. 4F). In particular, *Mtus1* and *Kcnk3* have been identified as reliable molecular markers in differentiation and thermogenic functions of human beige adipocytes^20^. In contrast, the expression of a classical brown adipocyte marker, *Zic1*, and fibroblasts marker genes, *Col1a2* and *Fsp1*, was reduced (Fig. 4G,H). These results support the conclusion that ciBAs are human brown (beige) adipocyte-like cells.

### Optimization of the induction period for ciBAs induced by RoFB in SFBAM

**Figure 5:**
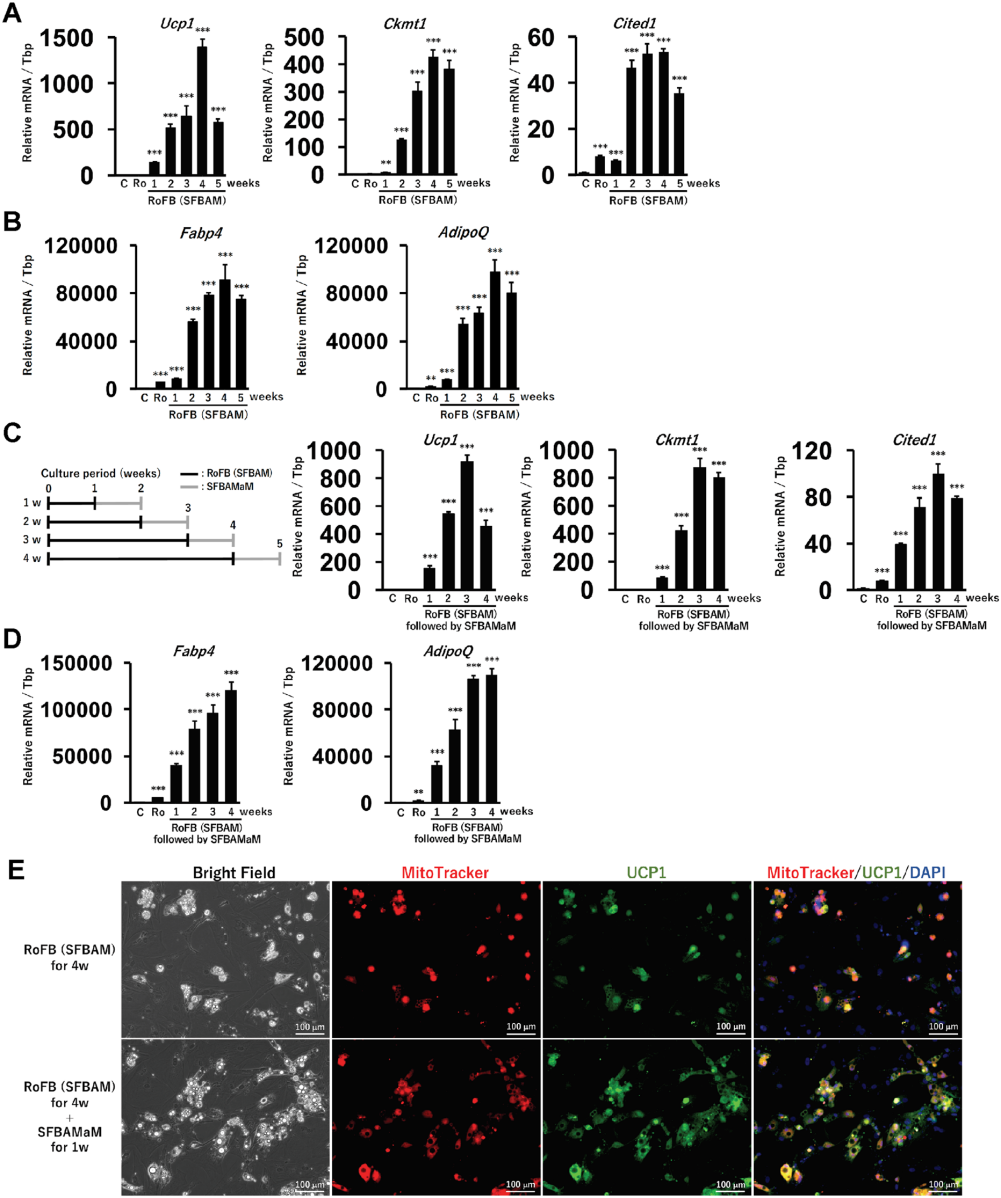
Optimization of period for ciBA induction by RoFB in SFBAM. (**A**) The expression of brown adipocyte-specific genes, *Ucp1*, *Ckmt1*, and *Cited1*, was quantified by qRT-PCR in ciBAs induced by RoFB in SFBAM from 1 to 5 weeks as indicated. “C” at the first lane represents the expression level of control fibroblasts treated with SFBAM only for 5 weeks. “Ro” at the second lane represents the expression level of the fibroblasts treated with Rosiglitazone only in SFBAM for 5 weeks. (**B**) qRT-PCR analyses of adipocyte-enriched genes, *Fabp4* and *AdipoQ*. (**C**) The expression of *Ucp1*, *Ckmt1*, and *Cited1*, was quantified in ciBAs induced by RoFB in SFBAM from 1 to 4 weeks as indicated, followed by incubation with the serum-free brown adipogenic maintenance medium, SFBAMaM, for another 1 week. In this experimental scheme, black bars represent the induction period with RoFB (SFBAM), while grey bars represent the maintenance step with SFBAMaM for 1 week after the induction. (**D**) qRT-PCR analyses of adipocyte-enriched genes, *Fabp4* and *AdipoQ*, were quantified. Data represent mean ± SD. Student’s t-test: **P < 0.01, ***P < 0.001. (**E**) Representative images of bright field, MitoTracker (red), UCP1 protein (green), and merged image in the ciBAs induced by either RoFB (SFBAM) for 4 weeks or RoFB (SFBAM) for 4 weeks followed by incubation with SFBAMaM for another 1 week. The nuclei were visualized by DAPI (blue). Scale bars represent 100 μm.

To optimize a conversion protocol for ciBAs induction with RoFB and SFBAM, the cells were harvested every week up to the 5th week. During the conversion, bright-field images indicate that number of the cells with lipid droplets was gradually increased (Supplementary Fig. S4A). The brown adipocyte-specific genes such as *Ucp1*, *Ckmt1*, and *Cited1* were upregulated from the incubation for 1 week, and this reached its maximum in ciBAs incubated for 4 weeks (Fig. 5A). The expression of adipocyte-enriched genes, *Fabp4* and *AdipoQ*, was also the highest in the incubation for 4 weeks (Fig. 5B). The incubation over 4 weeks no longer improved these expression levels. Next, ciBAs after the induction with RoFB in SFBAM were further incubated with SFBAMaM for another 1 week (Supplementary Fig. S4B). The expression levels of brown adipocyte-specific genes were the highest in ciBAs induced for 3 weeks followed by the maintenance step (Fig. 5C). The expression of *Fabp4* and *AdipoQ* was also the highest in the ciBAs induced for 3 and 4 weeks followed by the maintenance (Fig. 5D). Immunocytochemical analysis was performed after the incubation with RoFB in SFBAM for either 4 weeks or 4 weeks followed by an additional 1 week for the maintenance (Fig. 5E). The ciBAs after the maintenance step formed more lipid droplets compared to the ones without the maintenance. The lipid droplets, and clear signals of mitochondria and UCP1 proteins overlapped each other. These results suggest that ciBAs were converted within several weeks and that the lipid droplets, mitochondria levels, and UCP1 expression were properly maintained in SFBAMaM after the conversion.

To confirm availability of the combination of RoFB and SFBAM, two other lines of human dermal fibroblasts, HDF22 and HDF54, in addition to HDF38 were converted into ciBAs (Supplementary Table S1). Adipocyte-like cells with lipid droplets were successfully generated in all the three types of human fibroblasts, although the efficiency was variable (Supplementary Fig. S5A). All the cells exhibited the expression of *Ucp1* and *Fabp4* (Supplementary Fig. S5B,C). Immunocytochemical analysis indicated that the adipocytes derived from HDF38 and HDF54 showed increased levels of mitochondria and UCP1 proteins (Supplementary Fig. S6A). The conversion efficiencies were comparable for the fibroblasts of HDF38 and HDF54 (Supplementary Fig. S6B). These results suggested that the combination of RoFB in SFBAM enabled the direct conversion into ciBAs in the fibroblasts isolated from different subjects.

### Oxygen consumption rate (OCR) in ciBAs induced by RoFB in SFBAM

**Figure 6:**
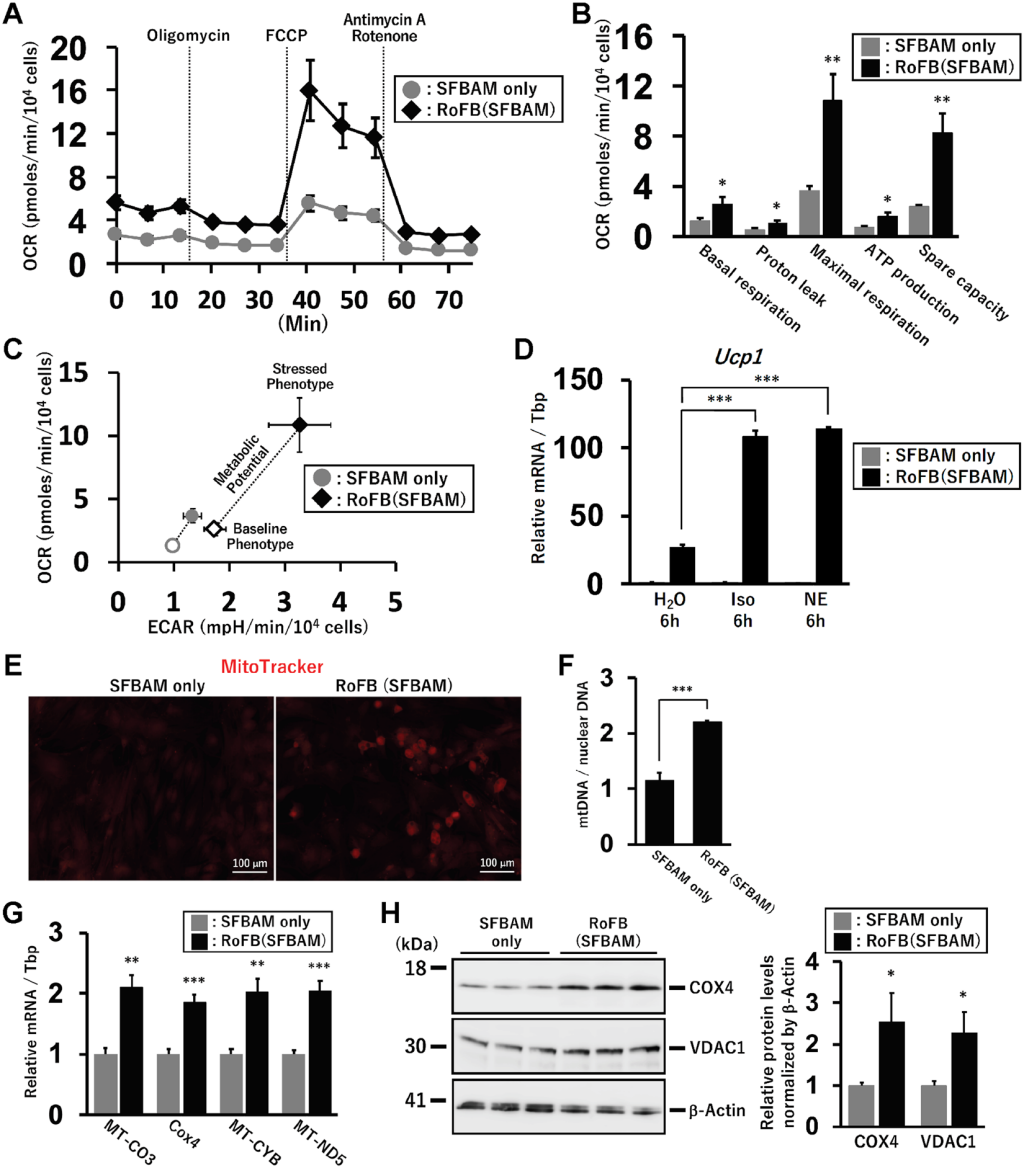
Increased oxygen consumption rate (OCR) in ciBAs induced by RoFB in SFBAM. (**A**) OCR was measured using the Flux analyzer in the control cells incubated with SFBAM only (grey circles) and ciBAs induced by RoFB in SFBAM (black diamonds). Oligomycin, FCCP, and Antimycin A/Rotenone were added during the measurement to final concentrations of 2, 0.3, and 0.5 μM, respectively. (**B**) The OCR corresponding to basal respiration, proton leak, maximal respiration, ATP production, and spare capacity was compared between the control and ciBAs. Data represent mean  ± SEM (n = 5). Student’s t-test: *P < 0.05, **P < 0.01. (**C**) Energy phenotype profile in the control cells (grey circles) and ciBAs (black diamonds). OCR and extracellular acidification rate (ECAR) were plotted under basal (open circle and diamond) and stressed condition (closed circle and diamond). Stressed condition was induced by the treatment with both Oligomycin and FCCP. The response to an induced energy demand under the stressed condition is represented as metabolic potential. (**D**) Induced expression of *Ucp1* mRNA was quantified in the control cells and ciBAs after treatment with either vehicle control (H_2_O), isoproterenol (Iso) at 1 μM, or norepinephrine (NE) at 2 μM for 6 hr. The ciBAs were induced by RoFB (SFBAM) for 3 weeks followed by SFBAMaM for 1 week. (**E**) Mitochondria were stained with MitoTracker in the control cells and ciBAs. Scale bars represent 100 μm. (**F**) Mitochondrial DNA levels were determined by qPCR analysis between the control and ciBAs. The DNA levels were normalized by nuclear DNA. (**G**) qRT-PCR analyses of mitochondrial marker genes, *MT-CO3*, *Cox4*, *MT-CYB*, and *MT-ND5*. (**H**) Mitochondrial marker proteins, COX4 and VDAC1, were quantified by immunoblotting. The band intensities were quantified and normalized by β-Actin loading control. Data represent mean ± SD. Student’s t-test: *P < 0.05, **P < 0.01, ***P < 0.001.

Elevated OCR is one of the most characteristic features for functional brown adipocytes. OCR in ciBAs induced by RoFB in SFBAM for 3 weeks was measured by Seahorse XFe96 extracellular flux analyzer (Fig. 6A). The OCR was typically varied during the measurement by pharmacological modulators for mitochondrial electron transport chain. Compared to the control cultured without RoFB combination, the OCR corresponding to basal respiration, proton leak, maximal respiration, ATP production, and spare respiratory capacity was significantly upregulated (Fig. 6B). In particular, the higher OCR for proton leak should be due to induced expression of UCP1. Energy phenotype profile plotting both OCR and extracellular acidification rate (ECAR) indicated that ciBAs had a higher metabolic potential under the stressed condition, which was totally different from the control cells (Fig. 6C). The treatment with β-adrenergic receptor agonists, either isoproterenol or norepinephrine, for 6 hr further induced the expression of *Ucp1* about 4 times (Fig. 6D), implying that β-adrenergic receptor pathway might be functional in ciBAs. Consistent with the observation that cellular mitochondrial signals were enhanced in ciBAs (Fig. 6E), enhanced mitochondrial DNA levels indicated that mitochondria biogenesis was promoted in ciBAs (Fig. 6F). The promoted mitochondria biogenesis in ciBAs was further supported by the increase of the expression level of both mitochondrial marker genes such as *MT-CO3*, *Cox4*, *MT-CYB*, and *MT-ND5* (Fig. 6G) and proteins, COX4 and VDAC1 (Fig. 6H). These results suggested that the ciBAs induced by RoFB in the serum-free medium also exhibited elevated oxidative metabolism along with increased mitochondria and UCP1 protein levels.

## Discussion

Previously, we successfully converted human dermal fibroblasts into brown adipocyte-like cells without the use of transgene^12^. Such a transgene-free direct conversion is a promising approach to rapidly prepare human brown adipocytes with a lower risk of tumorigenesis, which might be applicable for future clinical applications^1^. In this study, we have further simplified the combination of chemical cocktail consisting of Rosiglitazone, Forskolin, and BMP7 (RoFB) and developed the serum-free brown adipogenic medium (SFBAM). The combination of RoFB and SFBAM enabled an efficient conversion of human fibroblasts into ciBAs. We carefully checked the requirement of each component in the combination (Fig. 4). All the components in SMBAM as well as RoFB were effective for the direct conversion. Such simple and minimal formulations are of particular importance for convenient and reproducible preparation of ciBAs. In addition, recombinant human albumin without any animal-derived components as a substitute could allow SFBAM to be used as xeno-free and chemically defined medium, which more greatly expands the applications of ciBAs.

BMP signalling pathway and BMP7 play a critical role in rodent brown adipogenesis *in vivo*^21,22^. We identified that BMP7 treatment was also beneficial for the chemical conversion into ciBAs and mainly affected *Ucp1* expression rather than promoting the conversion itself in both FBS-containing and -free conditions. It should be noted that the effects were almost completely inhibited by BMP signalling inhibitors, LDN-193189 and Dorsomorphin, suggesting that BMP pathway activated by BMP7 is associated with adipocyte browning. In regard to TGF-β signalling pathway, it has been known that the activation dramatically inhibits adipogenesis and is involved in obesity-induced adipose tissue fibrosis^24,25^. In our previous report, the inhibitor, SB-431542, was one of the most effective compounds to drive the direct conversion in the FBS-containing medium^12^. However, SB-431542 was not required under the serum-free condition, probably because TGF-β pathway was already suppressed (Fig. 3). The observation implied that effects of small molecules on the direct conversion were dependent on culture conditions, and the repression of TGFβ signaling pathway was potentially required for the conversion into ciBAs.

In this study, both Rosiglitazone and Forskolin play a critical role in the direct conversion and *Ucp1* expression. Peroxisome proliferator-activated receptor gamma, PPARγ, activated by the selective agonist, Rosiglitazone, functions with other transcription factors and epigenetic regulators as a master transcription factor for adipogenesis^26^. Increased cellular levels of cAMP by Forskolin induce not only PKA-mediated phosphorylation of CREB but also P38 MAP kinase-mediated phosphorylation of ATF2^27^. The phosphorylated CREB and ATF2 directly bind to the promoter regions of *Ucp1* gene for the transcriptional activation.

Unexpectedly, T3 and Dexamethasone, agonists for two types of nuclear receptors, thyroid hormone receptor and glucocorticoid receptor, respectively, were almost essential for the direct conversion. It has been shown that glucocorticoids have complicated effects on development, proliferation, and metabolic functions of brown adipocytes^28^, while T3 positively regulates mitochondrial activity and biogenesis also in brown adipocytes^29^. Immunostaining of mitochondria and quantification of mitochondria DNA demonstrated that mitochondria biogenesis was promoted during the conversion into ciBAs. In addition, oxygen consumption rate (OCR) by mitochondria was increased in ciBAs (Fig. 6A,B), which is one of the most important characteristics in brown adipocytes to consume fatty acids and glucose for thermogenesis^18^. As shown in Fig. 6B, the increased rate of uncoupled respiration suggests that functional UCP1 is located across the inner mitochondrial membrane in ciBAs^30^.

Accumulated evidence has suggested that most brown adipocytes in adult human are derived from inducible beige adipocytes^31,32^. After the induction of ciBAs, loss of Rosiglitazone reduced *Ucp1* expression at least within 1 week (Supplementary Fig. S3C), while the adipocyte-like morphology with lipid droplets was largely preserved. This observation might be one of the characteristics of inducible beige adipocyte^33^. In addition, Myf5 is a myogenic lineage marker for classical brown adipocytes^18,22^, but the expression was not clearly activated in ciBAs. Thus our hypothesis is that ciBAs are more similar to beige adipocytes rather than classical brown adipocytes. This hypothesis is also supported by the fact that the expression of human beige adipocyte-enriched genes such as *Cited1*^34^, *Mtus1*, and *Kcnk3*^20^ was induced in ciBAs (Fig. 4E,F). According to a recent report that the expression level of a classical brown adipocyte marker, *Zic1*, was negatively associated with human BAT activity^35^, ciBAs significantly reduced the expression (Fig. 4G). Altogether, these findings suggest that the inducible and variable expression levels of *Ucp1* in ciBAs might be regulated by a molecular mechanism similar to the one involved in human adipocyte browning.

It is not feasible to consistently isolate pure primary beige adipocytes sporadically dispersed in white adipose tissue depots from different human subjects. Therefore, ciBAs induced by the serum-free condition could be a novel model for human beige adipocytes to reveal the molecular mechanism underlying adipocyte browning and to evaluate effects of small molecules on the browning. Such small molecules might have therapeutic potentials to enhance systemic energy metabolism not only by increasing the population of beige adipocytes in our body but also by activating the thermogenic functions through UCP1. The strategy might be a promising approach for the treatment of obesity and obesity-related metabolic diseases including diabetes mellitus and cardiovascular diseases. The development of the chemical compound-based direct conversion with the serum-free medium is the first step to simply and reproducibly provide ciBAs as a model for human beige adipocytes.

## Methods

### Direct conversion from human fibroblasts into ciBAs

Three lines of human dermal fibroblasts were purchased from DS Pharma Biomedical Co. (Osaka, Japan). The information is listed in Supplementary Table 1, and the fibroblasts derived from a human subject at age 38 years (HDF38) were mainly used. About 1.5 ×10^5^ cells were seeded on a 35-mm dish with high-glucose DMEM (11995-065, Gibco, MA, USA) supplemented with 10% FBS (HyClone, UT, USA) and penicillin/streptomycin (Gibco). After reaching 80-90% confluence of each type of human fibroblasts, the medium was changed to start direct conversion into ciBAs with FBS-containing adipogenic medium prepared from high-glucose DMEM (11995-065, Gibco) supplemented with 3,3’,5 Triiodothyronine (T3) (Sigma-Aldrich, MO, USA), Dexamethasone (Wako, Osaka, Japan), 3-isobutyl-1-methylxanthine (IBMX) (Wako), human recombinant insulin (Wako), L-ascorbic acid-2-phosphate (Sigma-Aldrich), 10% FBS (HyClone), and penicillin/streptomycin (Gibco). For the serum-free brown adipogenic medium (SFBAM), linoleic acid- and oleic acid-albumin (L9655-5ML, Sigma-Aldrich) was substituted for FBS. The final concentrations of these components are listed in Table 1. As described previously^12^, the chemical combination of 5CD-GM consists of Rosiglitazone (1 μM), SB-431542 (2 μM), LDN-193189 (1 μM), Dorsomorphin (1 μM), and Forskolin (7.5 μM). The combination RoFB consists of Rosiglitazone (1 μM), Forskolin (7.5 μM), and human recombinant BMP7 (20 ng/ml, 026-19171, Wako). For the direct conversion, human fibroblasts were incubated with the adipogenic medium either with or without the chemical cocktails for 3 weeks unless otherwise indicated. The medium was changed every 3 days. After the conversion, ciBAs were further cultured for the maintenance by the serum-free brown adipogenic maintenance medium (SFBAMaM). The formulation of SFBAMaM is listed in Table 2.

### qRT-PCR

Total RNA was extracted from the control fibroblasts and ciBAs induced by each experimental condition using FastGene RNA basic kit (Nippon Genetics, Tokyo, Japan). Reverse-transcription was performed by ReverTra Ace qPCR RT Master Mix with gDNA Remover (TOYOBO, Osaka, Japan). Real-time PCR analysis was performed using Power SYBR Green PCR Master Mix (Applied Biosystems, MA, USA). The reactions were carried out in triplicate and under the following conditions: 10 min at 95 °C, followed by 40 cycles of 15 sec at 95 °C and 60 sec at 60 °C. All the results were normalized by *Tbp* mRNA levels. The ratio of *Ucp1* to *Fabp4* mRNA was calculated as an indicator for adipocyte browning. The primer sequences for qRT-PCR have been described previously^12^. Additional primer sequences are listed in Supplementary Table S2. Unless otherwise indicated, the average of three biological replicates was calculated. The statistical significance was calculated by Student’s t-test.

### Quantification of mitochondrial DNA

Total genomic DNA was extracted with NucleoSpin Tissue (Takara, Shiga, Japan) from ciBAs induced by SFBAM including either no compound or the chemical cocktail, RoFB. mtDNA copy numbers were measured by qPCR using 10 ng total genomic DNA and Power SYBR Green PCR Master Mix. Each mtDNA was normalized to nuclear DNA level. Primer sequences for quantification of mitochondrial and nuclear DNA were as follows: mtDNA-Fwd, ACACCCTCCTAGCCTTACTAC; mtDNA-Rev, GATATAGGGTCGAAGCCGC; nuDNA-Fwd, AGGGTATCTGGGCTCTGG; NuDNA-Rev, GGCTGAAAAGCTCCCGATTAT^36^.

### Cell viability and cytotoxicity

Cell viability was measured by using Cell Counting Kit-8 (Dojindo, Kumamoto, Japan), according to the manufacturer’s instructions. The human fibroblasts (HDF38) were seeded on a 96-well plate and ciBAs were induced for 3 weeks as indicated. The cell viability was presented as a percent of control cells cultured in the medium only in parallel. To assess cytotoxicity, the activity of lactate dehydrogenase (LDH) released into the culture supernatants was measured by Cytotoxicity LDH Assay Kit-WST (Dojindo) according to the manufacturer’s instructions. Culture supernatants in ciBAs on a 96-well plate were transferred to a fresh 96-well plate for the assay.

### Immunoblot analysis

Immunoblot analysis was performed as previously reported^12^. Total proteins were extracted from ciBAs and the control fibroblasts with RIPA buffer [50 mM Tris–HCl (pH 8.0), 0.15 M sodium chloride, 0.5% sodium deoxycholate, 0.1% sodium dodecyl sulphate, 1% NP-40 substitute; Wako] and protease inhibitor cocktail (Wako). The proteins were subjected to 10% SDS-PAGE and transferred to a PVDF membrane (Thermo Fisher Scientific, DE, USA). The membranes were blocked with 5% skim milk followed by incubation with UCP1 antibody (MAB6158, R&D Systems, MN, USA) or β-Actin antibody (A5316, Sigma-Aldrich, MO, USA) at 4 °C overnight. Total SMAD2/3 proteins and phosphorylated SMAD2/3 proteins were detected by Smad2/3 (D7G7) XP Rabbit mAb (#8685, Cell Signalling Technology, MA, USA) and Phospho-Smad2 (Ser465/567)/Smad3 (Ser423/425) (D27F4) Rabbit mAb (#8828, Cell Signalling Technology), respectively. The membranes were incubated with either HRP-conjugated anti-rabbit or anti-mouse secondary antibody (Santa Cruz Biotechnology, CA, USA) for 1 hr at room temperature. Immunoreactive bands were detected by Immobilon Western Chemiluminescent HRP Substrate (Merck Millipore, Darmstadt, Germany). Each band intensity was quantified by densitometry using ImageJ software (National Institutes of Health).

### Immunostaining

Immunocytochemistry was performed as previously reported^11,12^. In brief, the cells were incubated with 250 nM MitoTracker Red CMXRos (Thermo Fisher Scientific) for 30 min at 37 °C in 5% CO_2_, according to the manufacturer’s instruction. Then the cells were fixed with 4% paraformaldehyde for 10 min. After washing with PBS, the cells were incubated with PBS containing 0.1% Triton X-100 for 5 min. They were blocked with PBS containing 3% skim milk for 1 hr at room temperature. The UCP1 antibody (ab10983, Abcam, Cambridge, UK) was diluted at 1/1000 with the blocking solution. The cells were incubated with the antibody overnight at 4 °C. After washing with PBS, the cells were further incubated with Alexa Fluor 488 donkey anti-rabbit IgG (Invitrogen, CA, USA) for 2 hrs at room temperature. Cell nuclei were stained with DAPI solution (Dojindo). All images were obtained using a BZ-X710-All-in-One Fluorescence Microscope (Keyence, Osaka, Japan) using a 20X objective lens (CFI Plan Fluor 20×, Nikon, Tokyo, Japan). All scale bars represent 100 μm. To evaluate the direct conversion efficiency, the numbers of DAPI-positive cells, the cells with lipid droplets, and UCP1-positive cells were counted from at least three different optical sections. The range of the number of cells was from 70 to 250 in each optical section.

### Measurement of OCR

For measurement of oxygen consumption rate (OCR) by mitochondria, human dermal fibroblasts (HDF38) were seeded on a 96-well plate and converted to ciBAs by RoFB in SFBAM for 3 weeks. As a control, the fibroblasts were cultured with the medium only in parallel. After incubation for 1 hr at 37 °C in a non-CO_2_ incubator, OCR was measured by the Seahorse XF96 Extracellular Flux Analyzer (Seahorse Bioscience Inc., MA, USA) according to the manufacturer’s instructions. During the analysis, Oligomycin, FCCP, and Antimycin A/Rotenone were added into each well via an injection apparatus to final concentrations at 2 μM, 0.3 μM, and 0.5 μM, respectively. Extracellular acidification rate (ECAR) was simultaneously measured.

### Statistics

All the results are presented as mean ± SD or SEM. Statistical analyses were performed by two-tailed Student’s t-test in the Excel (Microsoft) program. Statistical significance was defined as P-values < 0.05.

## Supplementary information

Supplemetary information.
