# Supplementary material for: A developed serum-free medium and an optimized chemical cocktail for direct conversion of human dermal fibroblasts into brown adipocytes
Source: Sci Rep. 2020 Feb 28;10:3775. doi: 10.1038/s41598-020-60769-x (PMC7048747; doi:10.1038/s41598-020-60769-x)
Supplement: Supplementary file 1 — Supplemetary information. [file 41598_2020_60769_MOESM1_ESM.pdf]

A developed serum-free medium and an optimized chemical cocktail for direct conversion of human dermal fibroblasts into brown adipocytes

Yukimasa Takeda[1] and Ping Dai[1]

[1]Department of Cellular Regenerative Medicine, Graduate School of Medical Science, Kyoto Prefectural University of Medicine, 465 Kajii-cho, Kawaramachi-Hirokoji, Kamigyo-ku, Kyoto 602-8566, Japan

Supplementary Information

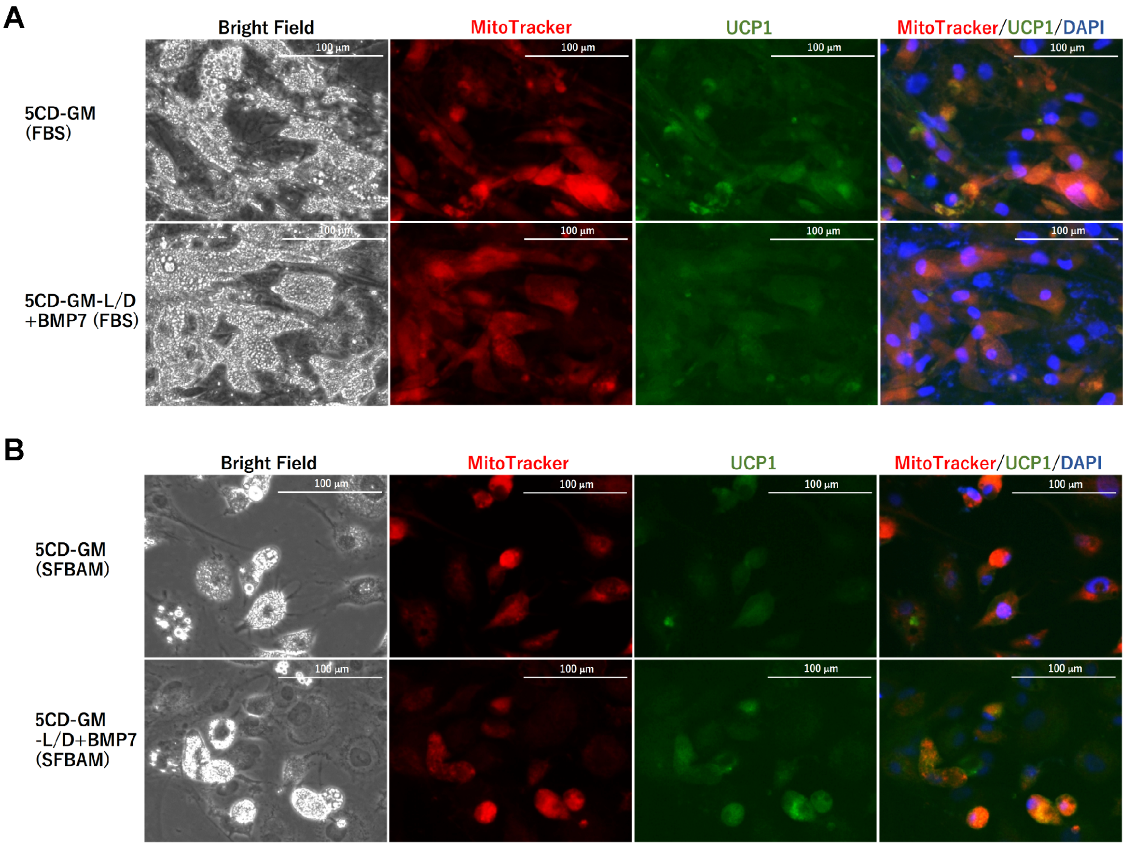

Figure S1. Higher magnification images in immunocytochemical analysis of ciBAs. (**A**) Representative images of bright field, mitochondrial labelling with MitoTracker (red), UCP1 protein expression (green), and merged image in ciBAs induced by either 5CD-GM or 5CD-GM-L/D+BMP7 in the FBS-containing medium. The nuclei were visualized by DAPI (blue). (**B**) Representative images of bright field, MitoTracker (red), UCP1 protein (green), and merged image in ciBAs induced by either 5CD-GM or 5CD-GM-L/D+BMP7 in SFBAM. The nuclei were visualized by DAPI (blue). Scale bars represent 100 µm.

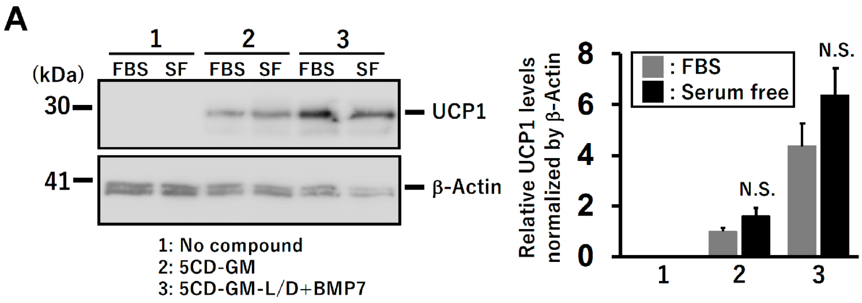

Figure S2. Western blotting analysis to quantify UCP1 protein levels in ciBAs induced by the FBS-containing and serum-free adipogenic medium.

(**A**) UCP1 protein levels were quantified by western blotting analysis. "FBS" represents the use of the adipogenic medium containing 10% FBS, while "SF" represents the use of the serum-free medium for the direct conversion into ciBAs. The band intensities were quantified by densitometry and normalized by β-Actin loading control. Data represent mean ± SD. Student's t-test: N.S.; not significant.

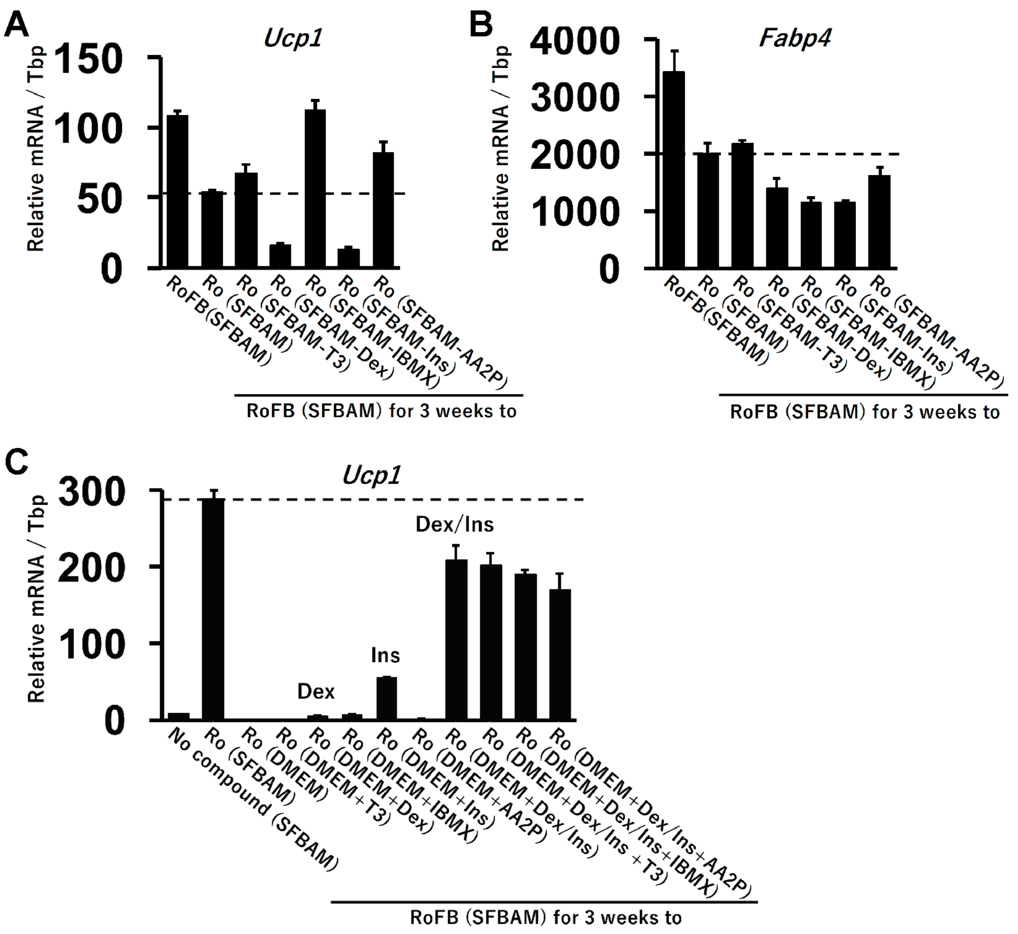

Figure S3. Optimization of components in serum-free brown adipogenic maintenance medium (SFBAMaM).

(**A,B**) qRT-PCR analyses of *Ucp1* (**A**) and *Fabp4* (**B**) expression in ciBAs induced by RoFB in SFBAM for 3 weeks followed by the medium from which each component was removed as indicated for the maintenance. (**C**) Optimization of components of the maintenance medium after the induction of ciBAs. Each single component was added to DMEM-based serum-free medium (DMEM) only supplemented with fatty acids-binding albumin and antibiotics. In addition, each of T3, IBMX, and L-ascorbic acid 2-phosphate (AA2P) was added to the DMEM-based medium supplemented with Dexamethasone (Dex) and Insulin (Ins). Data represent mean±SD.

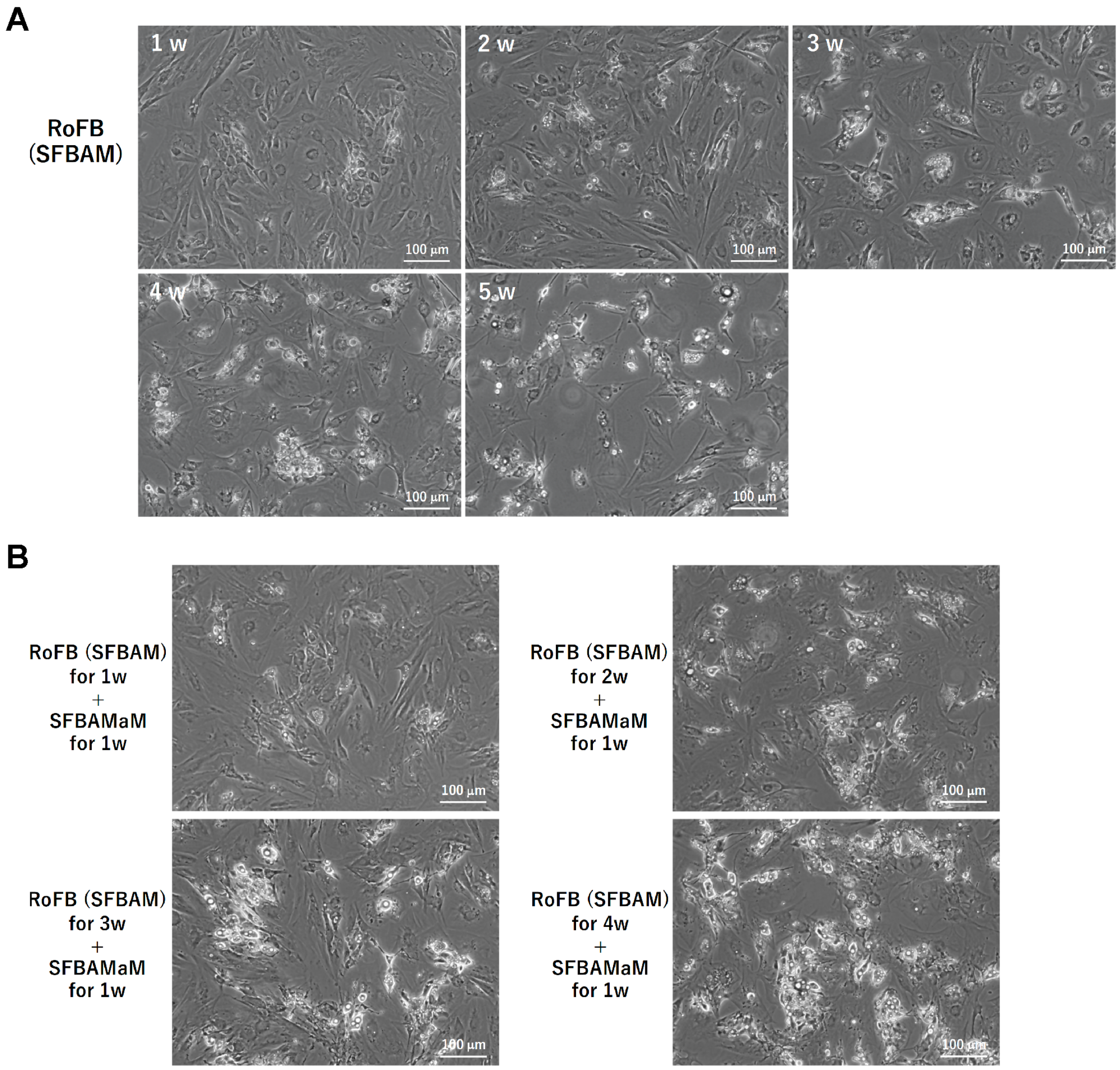

Figure S4. Representative images during the direct conversion into ciBAs with RoFB in SFBAM.

(**A**) Bright-field images of ciBAs induced by RoFB in SFBAM from 1 to 5 weeks as indicated. Scale bars represent 100 μm. (**B**) Representative images of ciBAs maintained by SFBAMaM for another 1 week after incubation with RoFB in SFBAM from 1 to 4 weeks as indicated.

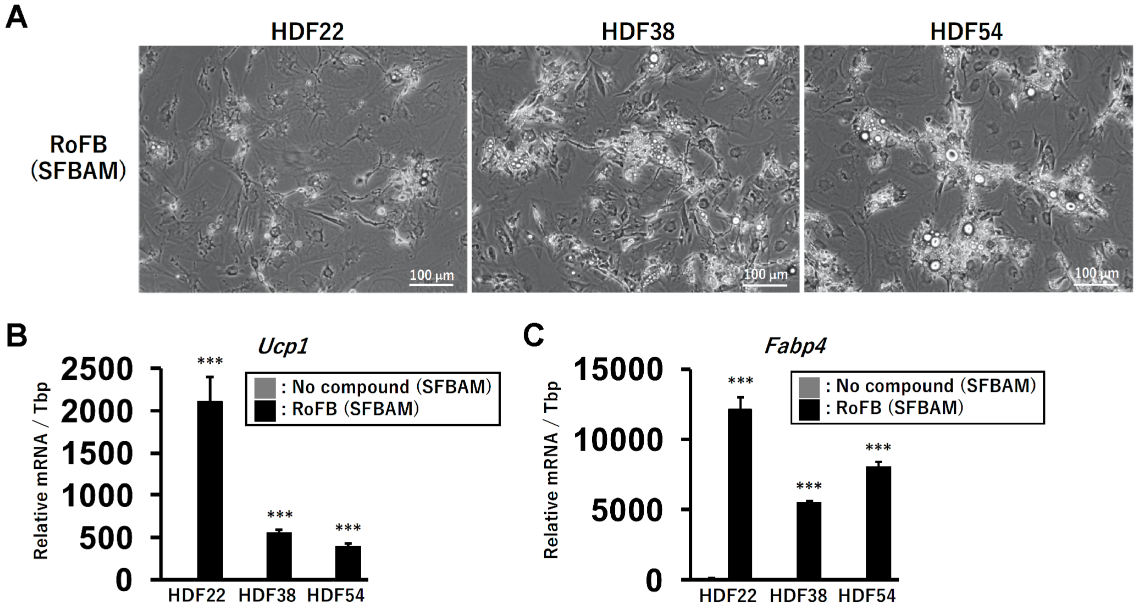

Figure S5. The direct conversion into ciBAs from different human dermal fibroblasts by the serum-free medium.

(**A**) Bright-field images in ciBAs derived from three types of human dermal fibroblasts, HDF22, HDF38, and HDF54. These fibroblasts were converted to ciBAs by RoFB in SFBAM for 3 weeks. Scale bars represent 100 μm. (**B,C**) qRT-PCR analyses of *Ucp1* (**B**) and *Fabp4* (**C**) expression in the ciBAs derived from HDF22, HDF38, and HDF54. Data represent mean ± SD. Student's t-test: ***P<0.001.

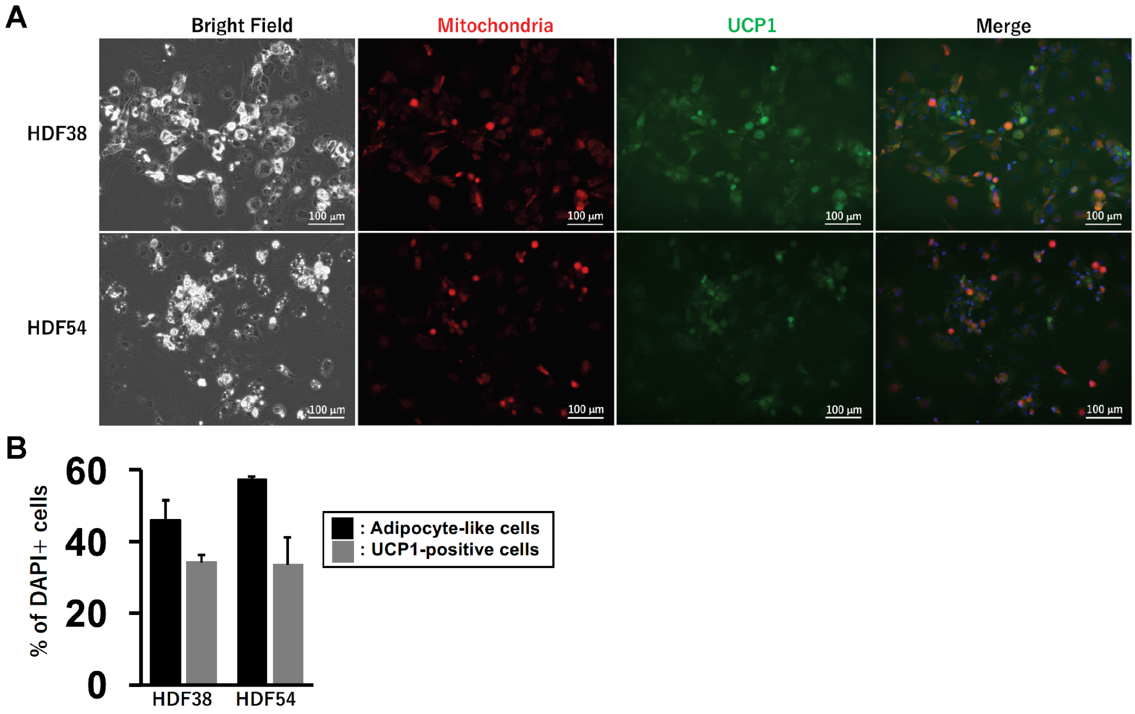

Figure S6. Immunocytochemical analysis of ciBAs derived from human dermal fibroblasts, HDF38 and HDF54.

(**A**) Representative images of bright field, MitoTracker (red), UCP1 protein (green), and merged image in ciBAs induced by RoFB in SFBAM for 3 weeks from the fibroblasts, HDF38 and HDF54. The nuclei were visualized by DAPI (blue). Scale bars represent 100 µm. (**B**) To evaluate the conversion efficiency, the percent ratios of adipocyte-like cells and UCP1-positive cells were calculated. Data represent mean ± SD.

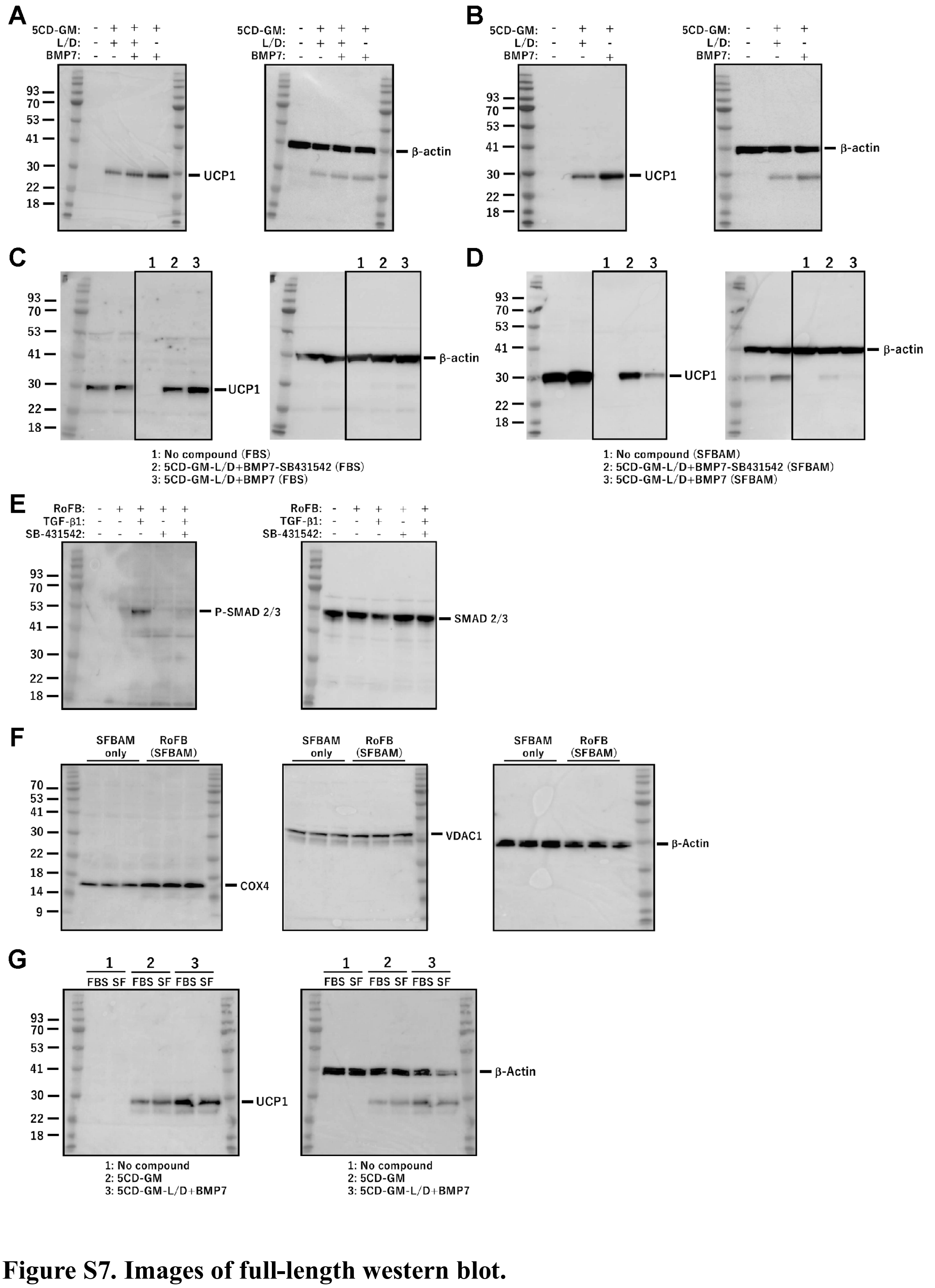

Figure S7. Images of full-length western blot.

(**A-G**) The raw results of western blot analysis are shown for Figure 1D (**A**), Figure 2D (**B**), Figure 3A (**C**), Figure 3B (**D**), Figure 3E (**E**), Figure 6H (**F**), and Supplementary Figure S2A (**G**).

Table S1. Information on human fibroblasts used for the direct conversion into ciBAs.

Abbreviation	Lot#	Passage	BMI	Age	Gender	Site
HDF22	DFM062509	3	29	22	Male	Breast
HDF38	DFM090214A	3	23.1	38	Male	Abdomen
HDF54	DDFM052010B	3	21.3	54	Female	Abdomen

Table S2. Sequences of primers used in qRT-PCR.

Gene	Sense primer	Antisense primer
Mtus1	TGAGGCGGAACAGTGACAATAG	ACCCATGACGACTGTGCAG
Kcnk3	CTCCTTCTACTTCGCCATCAC	AGAACACCTTGCCGCCATC
Bmp8b	AACAGGACCCTCCACGTCAG	GCTCGGAGCGTCTGAAGATC
Pgc1α	AGGTCAAGATCAAGGTCTCCAG	GGTGTCTGTAGTGGCTTGACTC
Fgf21	CTGCAGCTGAAAGCCTTGAAG	GTATCCGTCCTCAAGAAGCAG
Prdm16	AAGGGCAAGGAGCGATACAC	CCTCAGGTGTCTGGTGAGATTG
Cebpα	AACATCGCGGTGCGCAAGA	AGCTCCAGCACCTTCTGCT
Cebpβ	ACAAGGTCCTGGAGCTCAC	AAGTTCCGCAGGGTGCTGA
Cidea	AAGGCCACCATGTATGAGATGTAC	ACAGGAACCGCAGCAGACTC
Plin1	AGCATTGAGAAGGTGGTGGAG	ACTTCTGGGCTTGCTGGTG
Lipe	CTCAGTGTGCTCTCCAAGTG	CTTTCTGGTCTGAGTTGGAGTG
Fasn	CCTTCGAGGTGTCAGAGAAC	TTTCCGGGTGGTCGAAGAG
Elovl3	TTCTGGTCCTGGGTCTTTCTTC	CGCTTACGCAGGATGATGAAG
Zic1	GATGTGCGACAAGTCCTACAC	GAGGATTCGTAGCCAGAGCT
Myf5	AGCAGTACTTTTGACAGCATCTAC	GCAATCCAAGCTGGATAAGGAG
Fsp1	GGAGCTGCCCAGCTTCTTG	CTGTTGCTGTCCAAGTTGCTC
MT-CO3	CTCGCATCAGGAGTATCAATCAC	AGGAGGGTAAAATAGAGACCCAGT
Cox4	GCCAGAAGGCATTGAAGGAG	CATCTCAGCAAAGCTCTCCTTG
MT-CYB	ACTCCACCTCCTATTCTTGCAC	TGTGTAGTAAGGGTGGAAGGTG
MT-ND5	CTTAGGCGCTATCACCACTCTG	CTTGAAGTGGAGAAGGCTACGA
